# Supplementary material for: Nonmetallic Active Sites on Nickel Phosphide in Oxygen Evolution Reaction
Source: Nanomaterials (Basel). 2022 Mar 29;12(7):1130. doi: 10.3390/nano12071130 (PMC9000227; doi:10.3390/nano12071130)
Supplement: Supplementary file 1 [file nanomaterials-12-01130-s001.zip › nanomaterials-1653254-SI/manuscript-supplementary/nanomaterials-1653254-SI.pdf]

nanomaterials

Supplementary Materials

Nonmetallic Active Sites on Nickel Phosphide in Oxygen Evolution Reaction

Pengfei Zhang †, Hongmei Qiu †, Huicong Li, Jiangang He, Yingying Xu * and Rongming Wang *

Beijing Advanced Innovation Center for Materials Genome Engineering, Beijing Key Laboratory for Magneto-Photoelectrical Composite and Interface Science, School of Mathematics and Physics, University of Science and Technology Beijing, Beijing 100083, China; zpf3516@163.com (P.Z.); hmqiu@ustb.edu.cn (H.Q.);18166758766@163.com (H.L.); jghe2021@ustb.edu.cn (J.H.)

* Correspondence: xuyingying@ustb.edu.cn (Y.X.); rmwang@ustb.edu.cn (R.W.)

† These authors contributed equally to this work.

The details of OER calculations[1]

The relationship between Gibbs free energy (μ or G) and DFT energy (E_{DFT}) are constructed based on the following thermodynamic approximations. In all equations, μ_{H+}, μ_{e-}, μ_{H2}, μ_{O2} and μ_{H2O} denote chemical potentials of proton, electron, hydrogen molecule, oxygen molecule and water molecule, respectively. The superscript ^{0} is standard conditions of pH = 0 ,P=1bar and T = 298.15 K. E_{DFT} is the energy obtained from DFT calculations.

First of all, hydrogen electrode is assumed to be in equilibrium:

$$H^+(aq) + e^- \rightleftharpoons \frac{1}{2}H_2(g)$$

Thus

$$\mu^0_{H+} + \mu^0_{e-} = \frac{1}{2}\mu^0_{H2(g)} \qquad (S1)$$

Secondly, the experimental free energy increase for the following reaction is 2.46 eV:

$$H_2O(l) \rightleftharpoons \frac{1}{2}O_2(g) + H_2(g)$$

So,

$$\mu^0_{H2(g)} + \frac{1}{2}\mu^0_{O2(g)} - \mu^0_{H2O(l)} = 2.46\ [eV] \qquad (S2)$$

By definition,

$$G = H - TS \qquad (S3)$$

where G is Gibbs free energy which can also be represented by chemical potential μ, H is the enthalpy of the system which equals to E_{DFT} corrected by the zero-point energy (ZPE). S is the entropy of the system. The ZPS and entropy at 298 K.

As a result,

$$\mu = E_{DFT} + ZPE - TS \qquad (S4)$$

For H_2 molecule at standard conditions,

$$\mu^0_{H+} + \mu^0_{e-} = \frac{1}{2}\mu^0_{H2(g)} = \frac{1}{2}(E^{H2}_{DFT} + ZPE_{H2(g)} - TS^0_{H2(g)}) \qquad (S5)$$

For H_2O molecule in liquid phase,

$$\mu^0_{H2O(l)} \approx \mu_{H2O(g)} = E^{H2O(g)}_{DFT} + ZPE_{H2O(g)} - TS^0_{H2O}(0.035\ bar) \qquad (S6)$$

In this scheme, the OER is assumed to consist of four elementary reaction steps(* denotes a surface site and *X represents an adsorbed X intermediate on the surface). Equations (1) to (4) are also employed for deriving ΔG_1^0 to ΔG_4^0.

(1) $H_2O + * \rightarrow *OH + H^+ + e^-$

$$\Delta G_1^0 = \mu_{HO*}^0 + \mu_{H+}^0 + \mu_{e-}^0 - \mu_*^0 - \mu_{H2O(l)}^0 - eU$$

$$\mu_{HO*}^0 = E_{DFT}^{*OH} + ZPE_{*OH} - TS_{*OH}^0$$

$$\mu_*^0 = E_{DFT}^*$$

$$\Delta G_1^0 = E_{DFT}^{*OH} - E_{DFT}^* + \frac{1}{2}E_{DFT}^{H2(g)} - E_{DFT}^{H2O(l)} + ZPE_{*OH} + \frac{1}{2}ZPE_{H2(g)} - ZPE_{H2O(l)} - TS_{*OH}^0 - \frac{1}{2}TS_{H2(g)}^0 + TS_{H2O(l)}^0 = E_{DFT}^{*OH} + \frac{1}{2}E_{DFT}^{H2(g)} - E_{DFT}^* - E_{DFT}^{H2O(l)} + (\Delta ZPE - T\,\Delta\,S)_1$$

(2) $*OH \rightarrow *O + H^+ + e^-$

$$\Delta G_2^0 = \mu_{O*}^0 + \mu_{H+}^0 + \mu_{e-}^0 - \mu_{HO*}^0 - eU$$

$$\mu_{O*}^0 = E_{DFT}^{O*} + ZPE_{O*} - TS_{O*}^0$$

$$\mu_{*OH}^0 = E_{DFT}^{*OH} + ZPE_{*OH} - TS_{*OH}^0$$

$$\Delta G_2^0 = E_{DFT}^{*O} - E_{DFT}^{*OH} + \frac{1}{2}E_{DFT}^{H2(g)} + ZPE_{*O} - ZPE_{*OH} + \frac{1}{2}ZPE_{H2(g)} - TS_{*O}^0 + TS_{*OH}^0 - \frac{1}{2}TS_{H2(g)}^0 = E_{DFT}^{*O} + \frac{1}{2}E_{DFT}^{H2(g)} - E_{DFT}^{*OH} + (\Delta ZPE - T\,\Delta\,S)_2$$

(3) $*O + H_2O \rightarrow *OOH + H^+ + e^-$

$$\Delta G_3^0 = \mu_{*OOH}^0 + \mu_{H+}^0 + \mu_{e-}^0 - \mu_{O*}^0 - \mu_{H2O(l)}^0 - eU$$

$$\mu_{*OOH}^0 = E_{DFT}^{*OOH} + ZPE_{*OOH} - TS_{*OOH}^0$$

$$\mu_{*O}^0 = E_{DFT}^{*O} + ZPE_{*O} - TS_{*O}^0$$

$$\Delta G_3^0 = E_{DFT}^{*OOH} - E_{DFT}^{*O} + \frac{1}{2}E_{DFT}^{H2(g)} - E_{DFT}^{H2O(l)} + ZPE_{*OOH} - ZPE_{*O} + \frac{1}{2}ZPE_{H2(g)} - ZPE_{H2O(l)} - TS_{*OOH}^0 + TS_{*O}^0 - \frac{1}{2}TS_{H2(g)}^0 + TS_{H2O(l)}^0 = E_{DFT}^{*OOH} + \frac{1}{2}E_{DFT}^{H2(g)} - E_{DFT}^{*O} - E_{DFT}^{H2O(l)} + (\Delta ZPE - T\,\Delta\,S)_3$$

(4) $*OOH \rightarrow O_2 + H^+ + e^-$

$$\Delta G_4^0 = \mu_{O2(g)}^0 + \mu_*^0 + \mu_{H+}^0 + \mu_{e-}^0 - \mu_{*OOH}^0 - eU$$

$$\mu_{O2}^0 = 4.92\ [eV] + 2\mu_{H2O(l)}^0 - 2\mu_{H2(g)}^0$$

$$\mu_{*OOH}^0 = E_{DFT}^{*OOH} + ZPE_{*OOH} - TS_{*OOH}^0$$

$$\Delta G_4^0 = E_{DFT}^{*} - E_{DFT}^{*OOH} + 2E_{DFT}^{H2O(l)} - \frac{3}{2}E_{DFT}^{H2(g)} - ZPE_{*OOH} + 2ZPE_{H2O(l)} - \frac{3}{2}ZPE_{H2(g)} + TS_{*OOH}^0 - 2TS_{H2O}^0 + \frac{3}{2}TS_{H2(g)}^0 + 4.92 = E_{DFT}^{*} + 2E_{DFT}^{H2O(l)} - \frac{3}{2}E_{DFT}^{H2(g)} - E_{DFT}^{*OOH} + (\Delta ZPE - T\,\Delta\,S)_4$$

in which E_{DFT}^{*}, E_{DFT}^{*OH}, E_{DFT}^{*O} and $E_{DFT}^{*OOH}$are the calculated DFT energies of the clean surface and surfaces with adsorbed *OH, *O, and *OOH, respectively. $E_{DFT}^{H2O(l)}$ and $\frac{3}{2}E_{DFT}^{H2(g)}$ are the calculated energies for the isolated gaseous molecules H_2O, and H_2, respectively. ΔE_{ZPE} and ΔS are the zero-point energy and the entropy correction values, respectively [2].

Calculation method of surface energy

The surface energy is calculated by using the following equations[3]:

$$E_{surf} = \frac{1}{2A}\left(E_{slab} - n_{Ni}\left(E_{bulk-Ni} + \Delta\mu_{Ni}\right) - n_P\left(E_{bulk-P} + \Delta\mu_P\right)\right)$$

$$E_{f,Ni_{12}P_5} = 12\Delta\mu_{Ni} + 5\Delta\mu_P$$

$$E_{surf} = \frac{1}{2A}\left(E_{slab} - n_{Ni}E_{Ni\ bulk} - n_P E_{P\ bulk} - \frac{E_{f,Ni_{12}P_5}}{12}n_{Ni} + \Delta\mu_P\left(\frac{5n_{Ni}}{12} - n_P\right)\right)$$

Where E_{slab} is the total energy of the surface model, $E_{Ni\ bulk}$ and $E_{P\ bulk}$ are the total energy per atom of the metal Ni and black P, $E_{f,Ni_{12}P_5}$ is the formation energy of $Ni_{12}P_5$, n_{Ni} and n_P are the amount of Ni and P atoms in the surface model, respectively, $\Delta\mu_P$ is the chemical potential of P, and A is the surface area of the surface model.

For all surface models, a 15 Å vacuum layer was used for surface isolation to prevent interactions between adjacent surfaces. The top and bottom surface layers are relaxed and the middle 2 to 3 layers of atoms are fixed to simulate the bulk structure.

Supplementary Figures and Tables

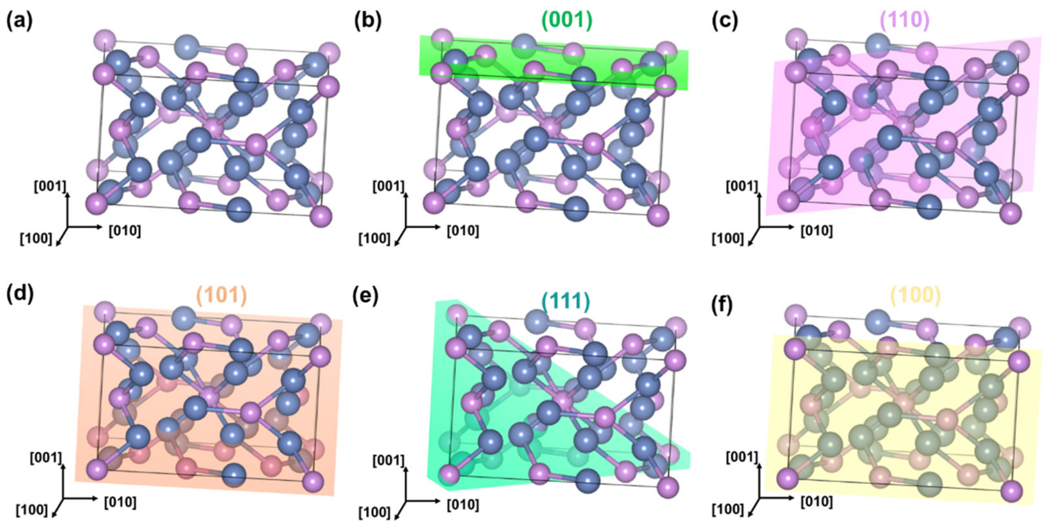

Figure S1. (a) $Ni_{12}P_5$ unit cell. (b)-(f) five lattice planes include (001), (110), (101), (111) and (100), presented by green, purple, orange, cyan and yellow, respectively.

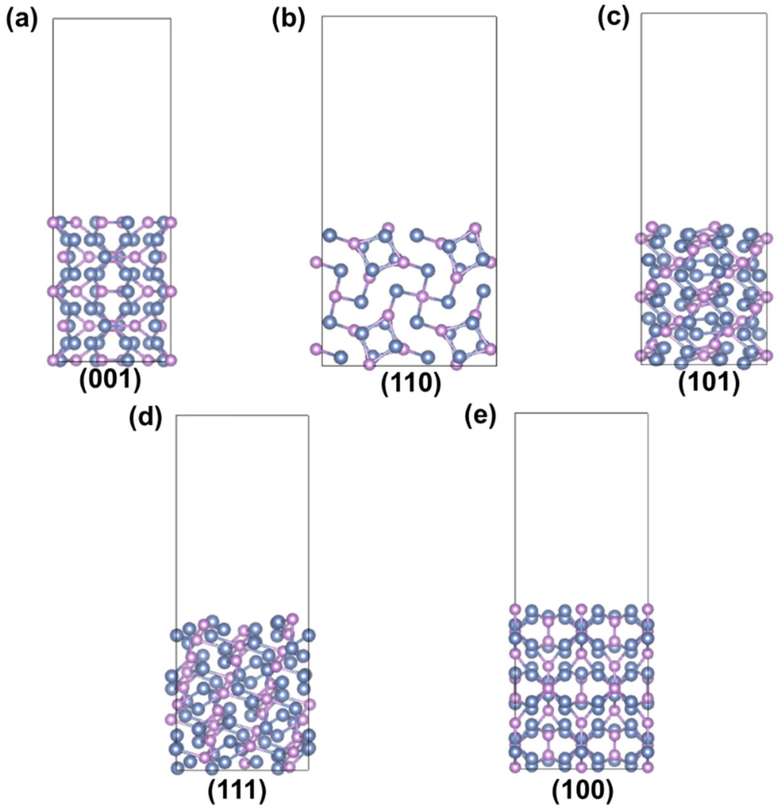

Figure S2. The terminated positions on different surfaces before optimization. (a) (001); (b) (110); (c) (101); (d) (111); (e) (100). Violet spheres stand for P atoms and blue spheres stand for Ni atoms.

Table S1. k-points setting for different structures.

Surface	k-points	Surface	k-points
Ni_2P (001)	3×3×1	Ni_2P (111)	3×4×1
$Ni_{12}P_5$(001)	4×4×1	$Ni_{12}P_5$(111)	5×4×1
Ni_5P_2(001)	3×3×1	Ni_5P_2(111)	3×3×1
Ni_3P (001)	4×4×1	Ni_3P (111)	3×4×1

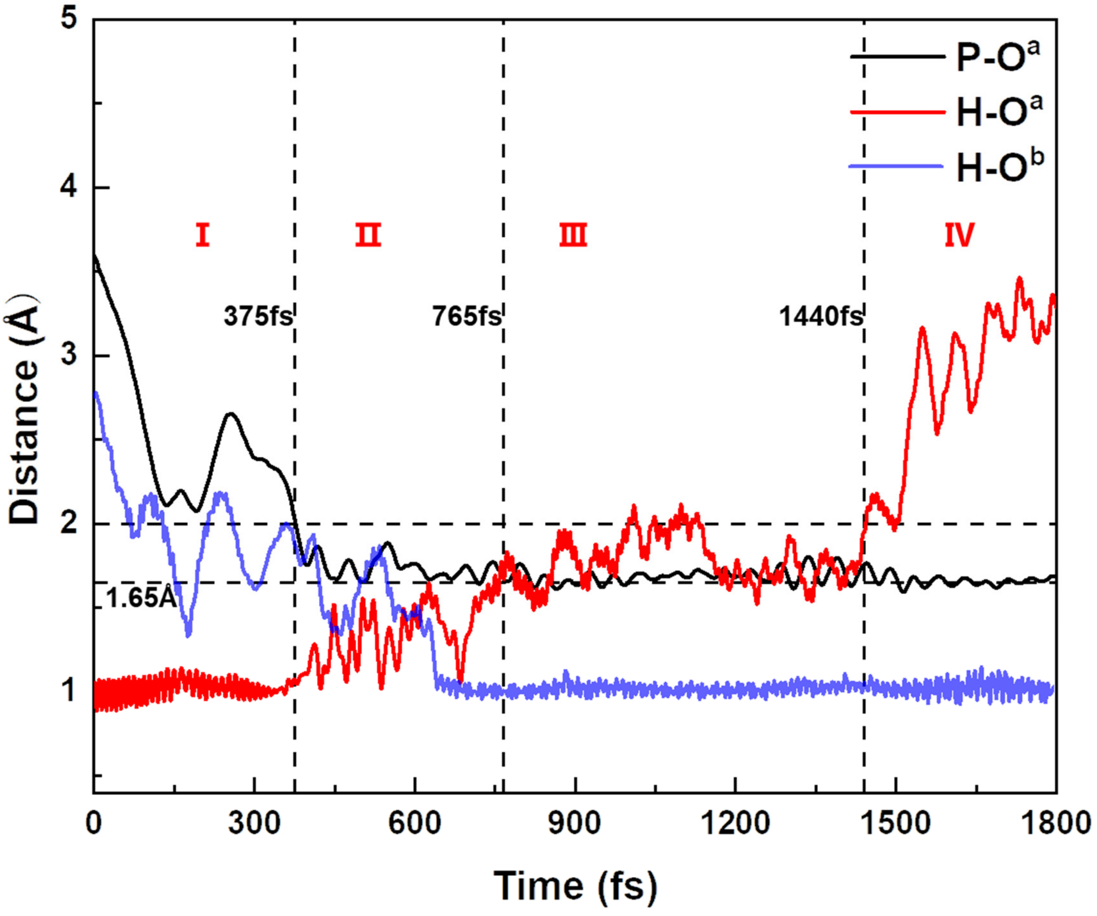

Figure S3. The time evolution of the key bond distances during the AIMD simulation. The black line is the bond distance between the P-O^a. The red line is the bond distance between the H-O^a. The blue line is the bond distance between the H-O^b. H is the dissociated H ion, O^a is the O atom in *OH; O^b is the O atom in another H_2O, and forms O^b-H…O^a with H.

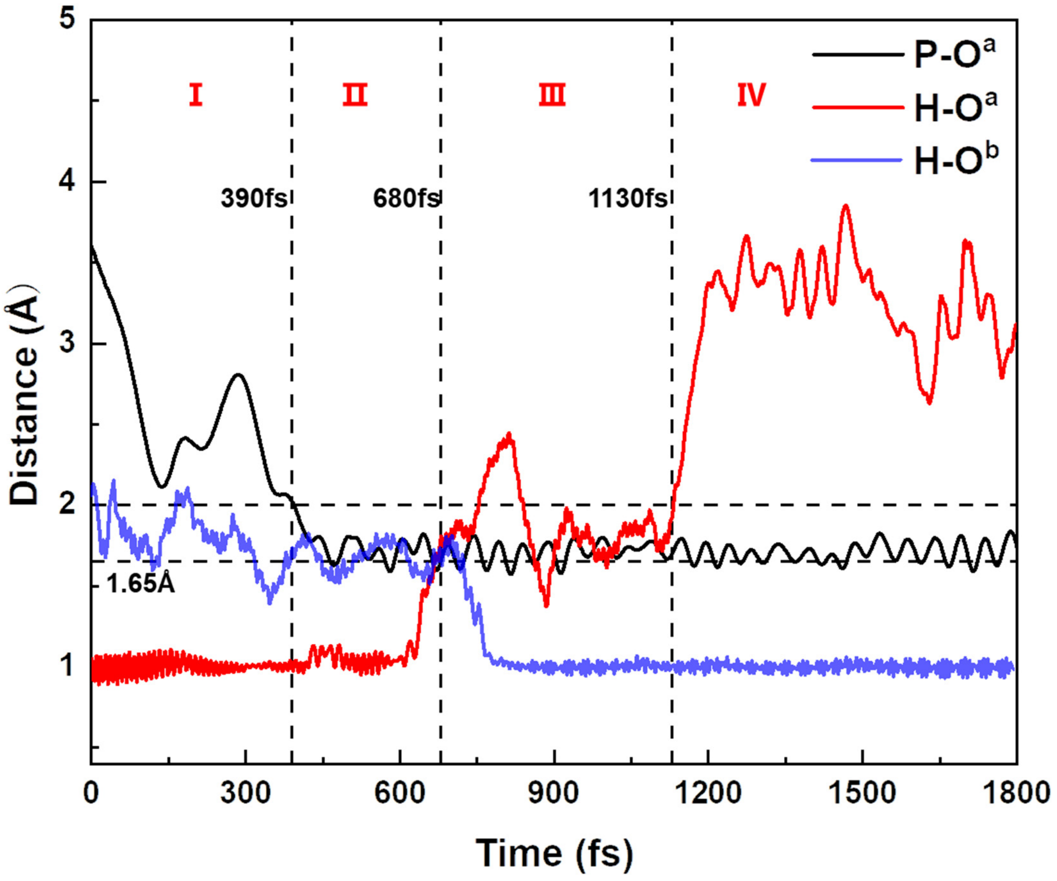

Figure S4. The time evolution of the key bond distances during the AIMD simulation. The black line is the bond distance between the P-O^a. The red line is the bond distance between the H-O^a. The blue line is the bond distance between the H-O^b. H is the dissociated H ion, O^a is the O atom in *OH; O^b is the O atom in another H_2O, and forms O^b-H…O^a with H.

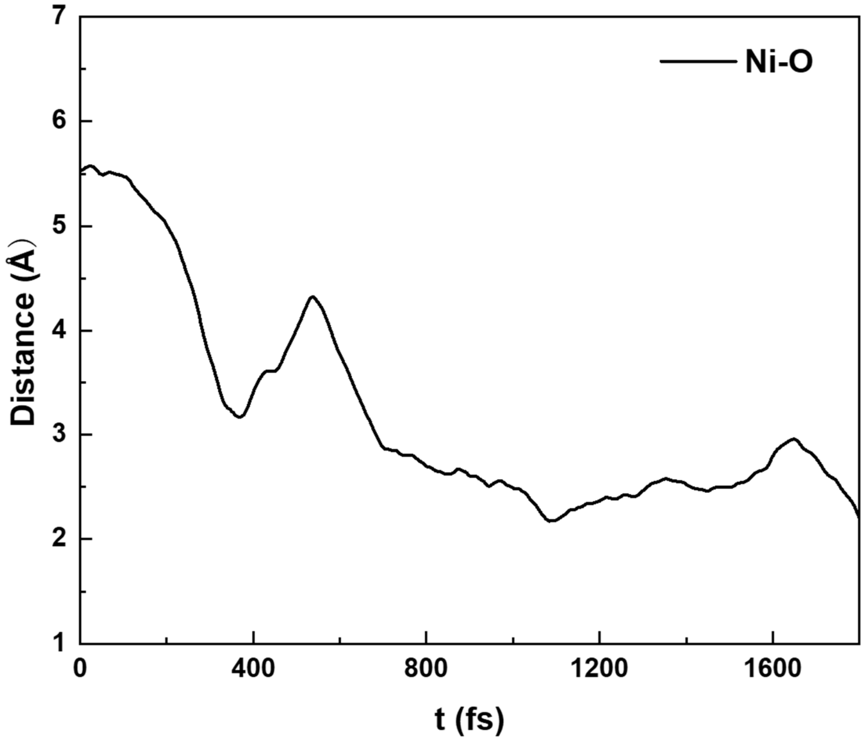

Figure S5. The time evolution of Ni-O distances during the AIMD simulation.

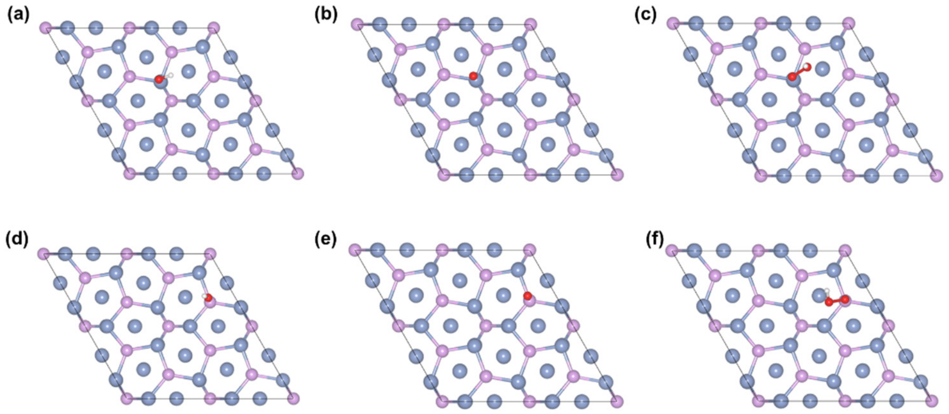

Figure S6. Geometric configuration of (a) Ni*OH, (b) Ni*O, (c) Ni*OOH, (d) P*OH, (e) P*O and (f) P*OOH adsorption on the Ni_2P (001) surface.

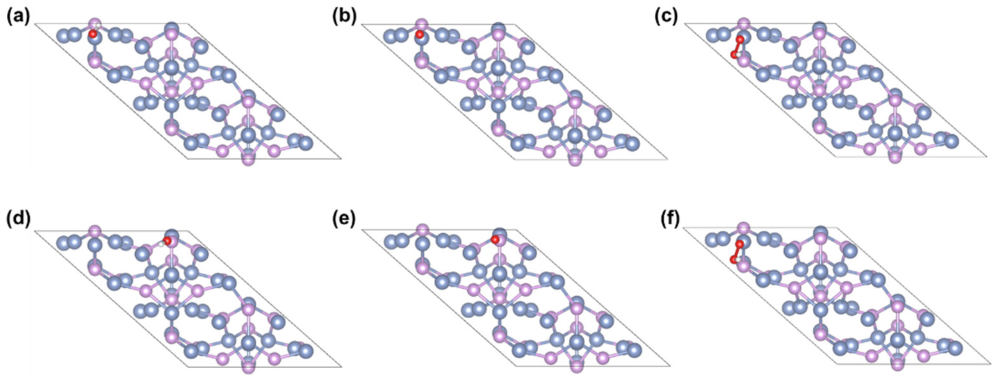

Figure S7 Geometric configuration of (a) Ni*OH, (b) Ni*O, (c) Ni*OOH, (d) P*OH, (e) P*O and (f) P*OOH adsorption on the Ni_2P (111) surface.

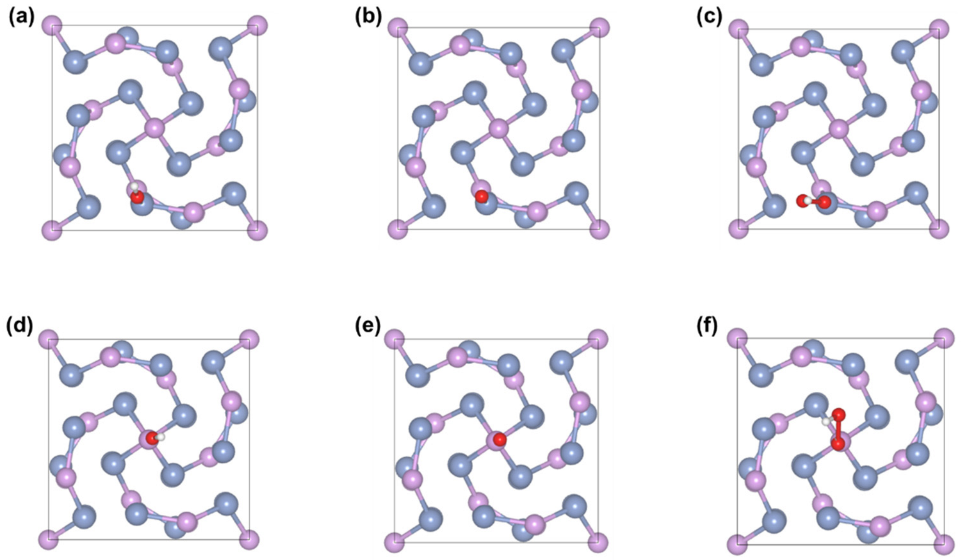

Figure S8. Geometric configuration of (a) Ni*OH, (b) Ni*O, (c) Ni*OOH, (d) P*OH, (e) P*O and (f) P*OOH adsorption on the $Ni_{12}P_5$ (001) surface.

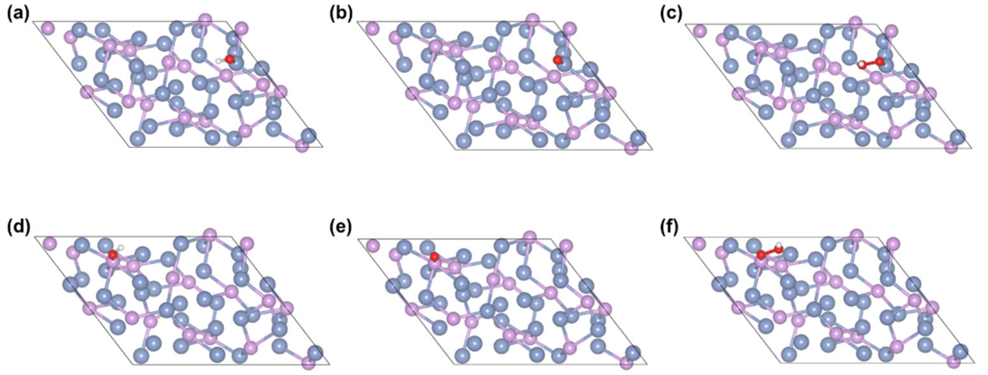

Figure S9. Geometric configuration of (a) Ni*OH, (b) Ni*O, (c) Ni*OOH, (d) P*OH, (e) P*O and (f) P*OOH adsorption on the $Ni_{12}P_5$ (111) surface.

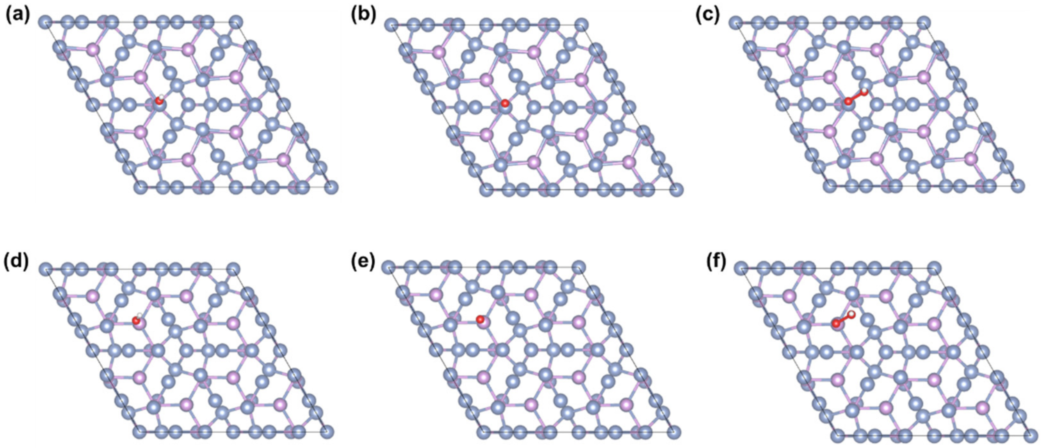

Figure S10. Geometric configuration of (a) Ni*OH, (b) Ni*O, (c) Ni*OOH, (d) P*OH, (e) P*O and (f) P*OOH adsorption on the Ni_5P_2 (001) surface.

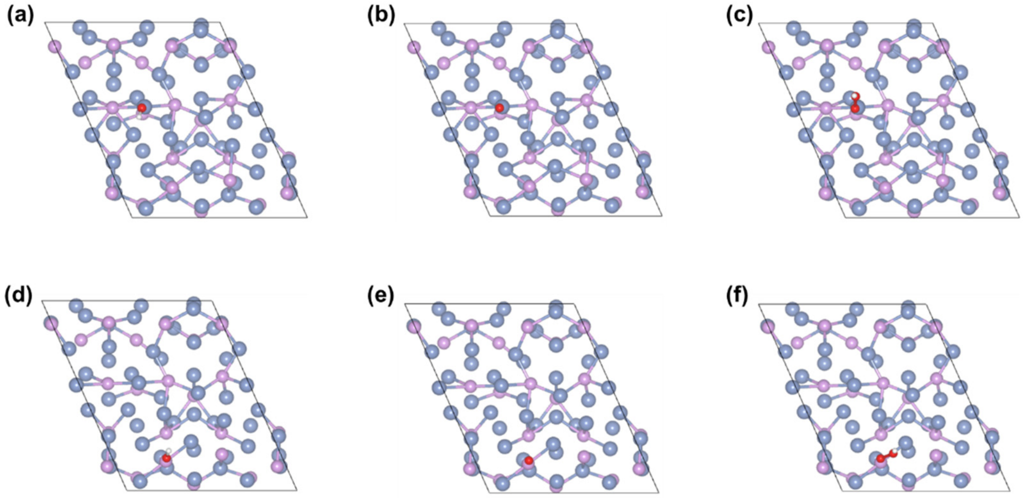

Figure S11. Geometric configuration of (a) Ni*OH, (b) Ni*O, (c) Ni*OOH, (d) P*OH, (e) P*O and (f) P*OOH adsorption on the Ni_5P_2 (111) surface.

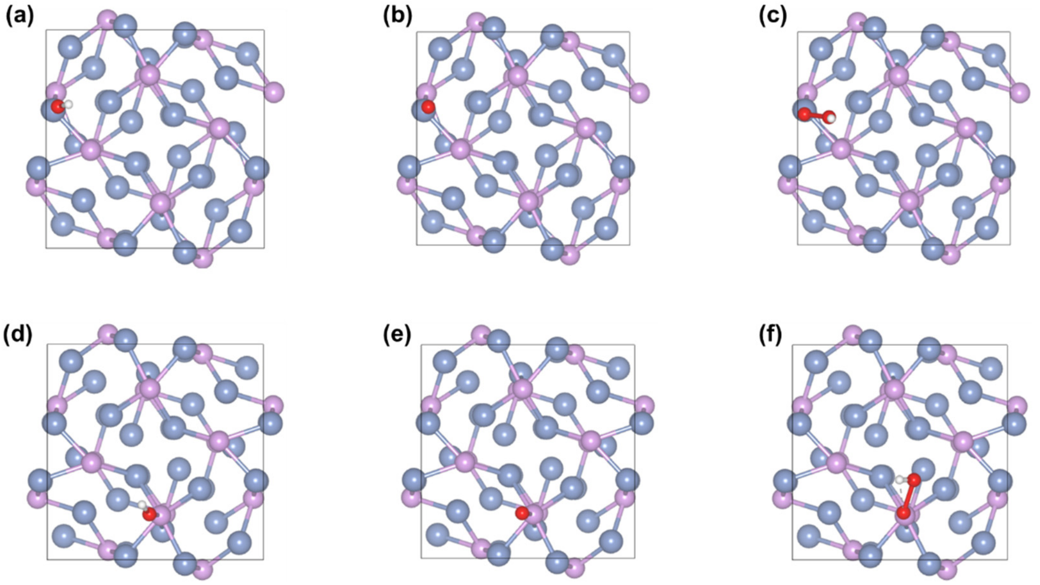

Figure S12. Geometric configuration of (a) Ni*OH, (b) Ni*O, (c) Ni*OOH, (d) P*OH, (e) P*O and (f) P*OOH adsorption on the Ni_3P (001) surface.

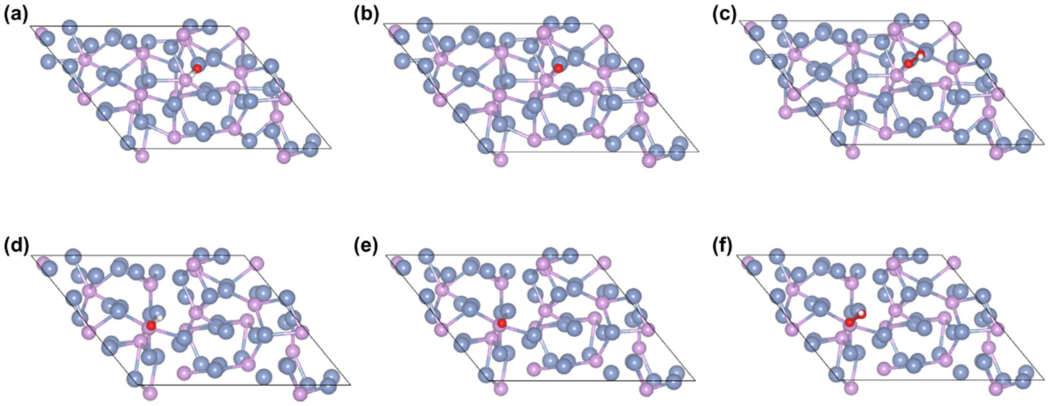

Figure S13. Geometric configuration of (a) Ni*OH, (b) Ni*O, (c) Ni*OOH, (d) P*OH, (e) P*O and (f) P*OOH adsorption on the Ni_3P (111) surface.

Table S2. The number of suspended bonds of P and Ni atoms on the exposed surface when establishing different nickel phosphide surfaces.

	**(001)**		**(010)**		**(100)**		**(101)**		**(011)**		**(110)**		**(111)**	
	**Ni**	**P**	**Ni**	**P**	**Ni**	**P**	**Ni**	**P**	**Ni**	**P**	**Ni**	**P**	**Ni**	**P**
**Ni_2P**	2,1	**3**	1	**3**	2,1	**3**	2	**3**	1	**3,1**	2,1	**4,3**	1	**3**
**$Ni_{12}P_5$**	0	**4,1**	1	**1**	1,0	**2**	1,0	**2,1**	1,0	**2,1**	1	**2**	1,0	**2,1**
**Ni_5P_2**	2	**3**	3,2	**3**	2,1	**2**	2,1	**2**	2,1	**2,1**	3,2,1	**3**	3,2,1	**3,2**
**Ni_3P**	2,1	**3**	2,1	**3**	2,1	**3**	3,2,1	**3**	2,1	**3**	2,1	**3**	3,1	**3**

Table S3. Comparison of OER catalytic performance of nickel phosphide in reference.

Catalyst	OER overpotential at 10 mA cm^{-2}	Ref.
$Ni_{12}P_5$	295mV	[4]
Ni_2P	330mV	[4-6]
Ni_3P	296mV	[7]
Ni_5P_2	300mV	[7]
Ni_5P_4	312mV	[8]

References:

1. Man, I.C.; Su, H.Y.; Calle-Vallejo, F.; Hansen, H.A.; Martínez, J.I.; Inoglu, N.G.; Kitchin, J.; Jaramillo, T.F.; Nørskov, J.K.; Rossmeisl, J. Universality in Oxygen Evolution Electrocatalysis on Oxide Surfaces. *ChemCatChem* **2011**, *3*, 1159-1165.
2. Rossmeisl, J.; Qu, Z.W.; Zhu, H.; Kroes, G.J.; Nørskov, J.K. Electrolysis of water on oxide surfaces. *Journal of Electroanalytical Chemistry* **2007**, *607*, 83-89.
3. Reuter, K.; Scheffler, M. Composition, structure, and stability ofRuO2(110)as a function of oxygen pressure. *Physical Review B* **2001**, *65*,
4. Menezes, P.W.; Indra, A.; Das, C.; Walter, C.; Göbel, C.; Gutkin, V.; Schmeiβer, D.; Driess, M. Uncovering the Nature of Active Species of Nickel Phosphide Catalysts in High-Performance Electrochemical Overall Water Splitting. *ACS Catalysis* **2016**, *7*, 103-109.
5. Wang, Q.; Zhang, Z.; Cai, C.; Wang, M.; Zhao, Z.L.; Li, M.; Huang, X.; Han, S.; Zhou, H.; Feng, Z.; et al. Single Iridium Atom Doped Ni2P Catalyst for Optimal Oxygen Evolution. *J Am Chem Soc* **2021**, *143*, 13605-13615.
6. Stern, L.-A.; Feng, L.; Song, F.; Hu, X. Ni2P as a Janus catalyst for water splitting: the oxygen evolution activity of Ni2P nanoparticles. *Energy & Environmental Science* **2015**, *8*, 2347-2351.
7. Zhang, P.; Lu, Y.R.; Hsu, C.S.; Xue, H.G.; Chan, T.S.; Suen, N.T.; Chen, H.M. Electronic structure inspired a highly robust electrocatalyst for the oxygen-evolution reaction. *Chem Commun (Camb)* **2020**, *56*, 8071-8074.
8. Lai, C.; Liu, X.; Deng, Y.; Yang, H.; Jiang, H.; Xiao, Z.; Liang, T. Rice-shape nanocrystalline Ni5P4: A promising bifunctional electrocatalyst for hydrogen evolution reaction and oxygen evolution reaction. *Inorganic Chemistry Communications* **2018**, *97*, 98-102.
